# Chondroitin sulfate alleviates osteoarthritis by upregulating HSPA8 to inhibit chondrocyte ferroptosis

**DOI:** 10.1371/journal.pone.0342242

**Published:** 2026-02-19

**Authors:** Jiayang Jiang, Yangyang Xu, Tianming Dai, Junyan Chen, Siming Li, Qingqi Meng

**Affiliations:** 1 Guizhou Medical University, Guiyang City, Guizhou Province, China; 2 Guangzhou Red Cross Hospital of Jinan University, Guangzhou, Guangdong Province, China; 3 The Fourth People’s Hospital of Guiyang, Guiyang City, Guizhou Province, China; The Affiliated Changzhou No 2 People's Hospital of Nanjing Medical University, CHINA

## Abstract

Osteoarthritis (OA) is a prevalent degenerative joint disease with no curative treatment currently available. Recent evidence suggests that chondrocyte ferroptosis contributes to OA progression. Chondroitin sulfate (CS), widely used in OA management, exhibits anti-inflammatory and antioxidant properties, yet its role in modulating ferroptosis remains unclear. In this study, we investigated whether CS alleviates OA by inhibiting chondrocyte ferroptosis and explored the underlying mechanisms. Using an in vitro ferroptosis model induced by RSL3 in rat chondrocytes, we found that CS significantly restored cell viability and ameliorated ferroptosis-related changes, including reduction of intracellular and mitochondrial ROS, lipid peroxidation, and iron overload. CS also downregulated the expression of ferroptosis markers PTGS2 and ACSL4, while upregulating SLC7A11 and HSPA8 in a dose-dependent manner. Network pharmacology and transcriptomic analysis identified HSPA8 as a key overlapping gene among CS targets, OA-related differentially expressed genes, and ferroptosis-related genes. In a rat OA model induced by modified Hulth surgery, CS treatment attenuated cartilage degradation, as evidenced by improved OARSI scores, restored COL2A1 expression, and suppressed MMP13. Immunohistochemistry confirmed that CS upregulated SLC7A11 and HSPA8 while downregulating ACSL4. These findings demonstrate that CS mitigates OA progression by inhibiting chondrocyte ferroptosis, potentially through upregulation of HSPA8 and subsequent enhancement of SLC7A11 expression. Our study provides novel insights into the mechanism of CS in OA treatment and highlights ferroptosis as a promising therapeutic target.

## Introduction

OA, a highly prevalent degenerative joint disorder characterized by complex multifactorial pathogenesis, imposes substantial socioeconomic burdens on patients and healthcare systems globally. This disabling condition predominantly affects the elderly population, with women exhibiting higher disability rates and disease prevalence compared to men [1,2]. OA is a whole-joint disorder primarily characterized by pain, involving structural alterations in articular cartilage, subchondral bone, joint capsule, ligaments, synovium, and periarticular muscles [3]. Articular cartilage is a layer of hyaline cartilage covering the ends of bones. It is composed of chondrocytes and the ECM. Chondrocytes maintain the balance between catabolism and anabolism, and since the ECM is produced by chondrocytes—the sole cell type within articular cartilage—their survival is thus crucial for joint stability [4,5].Research indicates that osteoarthritis is a multifactorial disease influenced by a combination of determinants, including gender, age, ethnicity, genetic predisposition, smoking habits, and localized mechanical risk factors [6]. Among these risk factors, inflammation is a well-established driver of disease pathogenesis and irreversible cartilage damage, while emerging evidence implicates iron overload as a potential contributor. [7–9]. Inflammation upregulates key degradative enzymes within articular cartilage, including matrix metalloproteinases (MMPs), a disintegrin and metalloproteinase with thrombospondin motifs (ADAMTS), and aggrecanases. This enzymatic activity triggers the degradation of the cartilage extracellular matrix (ECM), leading to subchondral bone dysfunction and synovitis, ultimately resulting in OA [10,11]. Similarly, studies demonstrate that iron overload disrupts cellular iron homeostasis, inducing elevated levels of reactive oxygen species (ROS) in chondrocytes, suppressing type II collagen synthesis, and promoting the upregulation of local pro-inflammatory mediators. Collectively, these effects indicate an association between iron overload and both the pathogenesis and progression of OA [12,13]. In 2012, Dixon et al. first described a novel form of regulated cell death distinct from other known mechanisms. This process, characterized by iron-dependent lipid peroxidation, was termed ferroptosis [14]. Ferroptosis, as the name implies, is intrinsically linked to iron accumulation. Excess iron drives ferroptosis primarily via the Fenton reaction, which generates ROS [15]. Iron serves as a pivotal cofactor for lipid peroxidation enzymes (e.g., LOXs, POR) and ROS-generating metabolic enzymes [16]. Concurrently, excessive lipid peroxidation constitutes another defining hallmark of ferroptosis [17]. Studies demonstrate that glutathione peroxidase 4 (GPX4) activity serves as the cornerstone of cellular antioxidant defense against peroxides. Consequently, higher GPX4 expression confers enhanced resistance to ferroptosis [18]. Although prostaglandin-endoperoxide synthase 2 (PTGS2/COX-2) is frequently upregulated in inflammatory contexts, its elevated expression serves as a specific biomarker for ferroptosis [19]. System Xc ⁻ , an amino acid transporter composed of SLC7A11 and SLC3A2, is primarily regulated by SLC7A11; its inhibition by compounds like erastin blocks cystine uptake, thereby depleting cysteine and glutathione to induce ferroptosis [20,21]. The susceptibility to ferroptosis is governed by the metabolic flux of PUFAs into phospholipids, a pathway initiated by ACSL4 activation of free PUFAs and completed by LPCAT3-mediated esterification. Consequently, ACSL4 serves as a key sensitizing marker for this process [17,22]. Ferroptosis—a distinct form of iron-dependent regulated cell death (RCD)—has sparked considerable research interest across multiple disease domains, including cancer, neurodegenerative disorders, tissue/organ injury, and inflammatory and infectious diseases [23]. The role of ferroptosis in OA remained unconfirmed until 2021, when Yao et al. first demonstrated through in vitro and in vivo experiments that inhibiting chondrocyte ferroptosis represents an effective therapeutic strategy for OA [24]. Emerging evidence indicates that sulfation modification suppresses ferroptosis and oxidative damage in hippocampal neurons [25]. Chondroitin sulfate—a sulfated glycosaminoglycan (GAG)—is recognized as a Symptomatic Slow-Acting Drug for Osteoarthritis (SYSADOA) [26]. Substantial evidence confirms CS possesses anti-inflammatory and antioxidant properties [27]. Mechanistically, CS counteracts interleukin-1β (IL-1β)-induced reduction of proteoglycans (PGs) in chondrocytes [28] and inhibits nuclear translocation of NF-κB, thereby diminishing inflammatory biomarkers [29]. Antioxidant effects include suppression of free radical generation [30] and chelation of transition metals (e.g., Cu²⁺ or Fe²⁺) via ionic interactions between its anionic groups and metal cations. This metal chelation inhibits Haber-Weiss and Fenton reactions that drive oxidative stress [31]. Furthermore, CS attenuates chondrocyte apoptosis by preserving mitochondrial integrity and downregulating caspase-3 and caspase-9 expression, consequently mitigating cartilage matrix degradation [32]. However, whether CS-mediated chondroprotection involves modulation of chondrocyte ferroptosis remains unknown. Building upon this evidence, we aim to investigate whether CS mitigates osteoarthritis progression by inhibiting chondrocyte ferroptosis and elucidate its underlying mechanisms. In this study, we established an in vitro ferroptosis model using RSL3-treated chondrocytes. Following CS intervention, we assessed changes in:

(i) cellular viability,
(ii) ferroptosis-related biomarkers, and
(iii) mitochondrial function to determine CS’s cytoprotective effects. These findings were subsequently validated in a rat OA model.

## Materials and methods

### Isolation and culture of rat chondrocytes

Primary chondrocytes were isolated from 2-week-old Sprague Dawley rats by dissecting articular cartilage from bilateral knee joints. The cartilage was minced into approximately 1 mm³ fragments and sequentially digested: first with 0.25% trypsin (25200072, Gibco, Grand Island, NY, USA) for 1 hour at 37°C under 5% CO₂ with continuous shaking, followed by 0.2% type II collagenase (C6885, Sigma, MO, USA) digestion under identical conditions for 4–6 hours. After centrifugation (300 × g, 5 min), the supernatant was discarded and the pellet was filtered through a 70-μm sterile strainer. Cells were resuspended in DMEM/F12 medium (C11330500BT, Gibco, Beijing, China) supplemented with 10% fetal bovine serum (A3160801, Gibco, Grand Island, NY, USA), 1% penicillin, and 1% streptomycin sulfate (15140122, Gibco, Grand Island, NY, USA). Isolated chondrocytes were cultured in a humidified incubator (37°C, 5% CO₂) for subsequent experiments.

### Toluidine blue staining

Chondrocyte morphology was observed using toluidine blue staining. Briefly, chondrocytes were seeded in 6-well plates until reaching 80% confluence, then co-treated for 4 hours with RSL3 (0.2μM; SML2234, Sigma, MO, USA) and either chondroitin sulfate (1 mg/ml or 3 mg/ml; C875624, Macklin, Shanghai, China) or Ferrostatin-1 (Fer-1) (0.1μM; F792503, Macklin). The culture medium was aspirated and cells were washed twice with PBS. An appropriate volume of toluidine blue staining solution (Y026912, Beyotime, Shanghai, China) was added to each well for 5-minute staining. An equal volume of distilled water was added and mixed, followed by 15-minute incubation without disturbance. After two washes with distilled water, samples were examined microscopically.

### Cell viability assay

Chondrocytes were seeded in 96-well plates (4,000 cells/well) and incubated overnight. The following day, after confirming cell adherence microscopically, treatment groups were established. The original medium was aspirated and replaced with RSL3, CS, or Fer-1 as previously described for 4-hour co-treatment. Post-treatment, cellular modeling status was examined microscopically. Drug-containing medium was then aspirated, and pre-prepared CCK-8 working solution (C0037, Beyotime; CCK-8 reagent:DF12 medium = 1:10) was added to each well. Plates were incubated at 37°C for 1–2 hours. Absorbance was measured at 450 nm using a microplate reader, with cell viability calculated based on optical density values.

### Measurement of intracellular iron

Chondrocytes were seeded in 12-well plates and cultured overnight. The supernatant was aspirated, followed by three washes with serum-free DF12 medium. After replacing with drug-containing medium and completing treatment, the medium was removed and cells were rinsed thrice with serum-free DF12. FerroOrange working solution (S1070S, Beyotime) was added according to manufacturer’s instructions, and cells were incubated at 37°C under 5% CO₂ for 30 minutes. Fluorescence microscopy was subsequently performed to examine intracellular iron accumulation.

### Measurement of adenosine triphosphate (ATP) and malondialdehyde (MDA)

Chondrocytes in 6-well plates were lysed with cell lysis buffer and centrifuged to collect supernatants. Samples were analyzed using appropriate working solutions according to manufacturer protocols for the MDA Assay Kit (S0131S, Beyotime) and Enhanced ATP Assay Kit (S0027, Beyotime). Absorbance at respective wavelengths was measured with a spectrophotometer. ATP and MDA levels were normalized to protein concentrations determined by the BCA Protein Assay Kit (P0012, Beyotime).

### Detection of intracellular reactive oxygen species (ROS) and lipid peroxidation (LPO)

Intracellular ROS and LPO levels were detected using fluorescent probes DCFH-DA (S0033S, Beyotime) and BODIPY 581/591 C11 (D3861, Invitrogen, CA, USA) according to manufacturer protocols. Chondrocytes were seeded in 24-well plates. After 24 hours, cells were treated with designated compounds for 4 hours. Wells were washed three times with PBS and incubated with 250 μL of diluted 10 μM DCFH-DA or 10 μM BODIPY 581/591 C11 at 37°C under dark conditions for 30 minutes. Following incubation, cells underwent three 3-minute PBS washes. Fluorescence microscopy was performed with DCFH-DA using 488 nm excitation/525 nm emission wavelengths, while oxidized BODIPY 581/591 C11 was detected at 500 nm excitation for green fluorescence quantification (oxidized state).

### RNA extraction and RT‑qPCR reaction

Total RNA was extracted from chondrocytes using TRIzol reagent (15596026CN, Invitrogen, CA, USA). cDNA synthesis was performed with PrimeScript RT Master Mix (RR036A, Takara, Beijing, China). RT-qPCR reactions were conducted using TB Green RT-PCR reagents (RR420A, Takara). Relative mRNA expression was normalized to GAPDH using the 2 − ΔΔCt method. Primer sequences are listed in S1 Table.

### Western blot analysis

Following 4-hour drug treatment in 6-well plates, chondrocytes were lysed using RIPA lysis buffer (P0013B, Beyotime) containing 1% protease inhibitor cocktail (P8340, Sigma). Lysates were collected and subjected to electrophoresis, followed by protein transfer to PVDF membranes (IPVH00010, Sigma). Membranes were incubated overnight at 4°C with primary antibodies. The next day, membranes were washed three times with TBST and incubated with secondary antibodies (1:5000 dilution; AP132P, AP124P, Sigma) for 2 hours. Protein bands were visualized using the SuperSignal West Pico chemiluminescent substrate (34580, Thermo Scientific, CA, USA) on a Bio-RAD ChemiDoc XRS+ system (Bio-RAD, CA, USA).

The primary antibodies: β-Tubulin (1:2000; 10094–1-AP, Proteintech),GAPDH (1:1000; ARG10112, Arigo, Taiwan, China), GPX4 (1:500; 30388–1-AP, Proteintech, Wuhan, China), SLC7A11 (1:1000; 26864–1-AP, Proteintech), COL2A1 (1:1000, 28459–1-AP, Proteintech), HSPA8 (1:2000, 10654–1-AP, Proteintech).

### Measurement of mitochondrial membrane potential (ΔΨm)

Chondrocytes were seeded in 24-well plates. After 24 hours, cells underwent 4-hour drug treatment. Culture medium was aspirated and replaced with 500 μl fresh medium. Subsequently, 1 ml JC-1 staining working solution (C2003S, Beyotime) was added and gently mixed. Following 20-minute incubation at 37°C, supernatant was removed and cells were washed three times with JC-1 staining buffer. Finally, 500 μl culture medium was added prior to fluorescence microscopy imaging. Quantitative analysis was performed using ImageJ software to calculate red/green fluorescence ratios.

### Detection of mitochondrial superoxide (MitoSOX)

Chondrocytes were seeded in 24-well plates. After 24-hour incubation, cells underwent 4-hour drug treatment. Culture medium was aspirated, and cells were stained with 300 μl per well of 3 μM MitoSOX™ Red reagent (diluted in PBS; M36008, Invitrogen) containing Hoechst 33342 nuclear counterstain (C1011, Beyotime; 1:300 dilution in PBS). Plates were incubated at 37°C for 10 minutes protected from light. Fluorescence images were acquired using microscopy and quantified with ImageJ.

### Network pharmacology

The molecular structure of CS was retrieved from the NCBI database. Potential therapeutic targets were predicted using PharmMapper. Identified target genes were imported into the STRING platform to construct a protein-protein interaction (PPI) network. Functional enrichment analysis was performed through Gene Ontology (GO) and Kyoto Encyclopedia of Genes and Genomes (KEGG) databases, with target proteins evaluated across biological processes (BP), cellular components (CC), and molecular functions (MF). Results were visualized via bar plots and bubble plots.Osteoarthritis transcriptomic data were obtained from the GEO database. Differential gene expression analysis was conducted using the limma package in R. Differentially expressed genes (DEGs: The filtering criteria for identifying DEGs were an adjusted p-value (adj.P.Val < 0.05) and an absolute log2 fold-change (|logFC| > 1).) were visualized through heatmaps generated with the pheatmap function and volcano plots created using the ggplot2 package. Finally, overlapping genes between CS targets and OA DEGs were identified using the VennDiagram package, with intersection results visualized and exported.

Finally, the intersection among four gene sets—CS putative targets, OA-DEGs, ferroptosis-related candidates retrieved from GeneCards, and experimentally validated ferroptosis regulators archived in FerrDb v3—was determined by means of the VennDiagram R package. The resulting overlapping genes were visualized and exported as a high-confidence consensus list for subsequent functional enrichment and network analyses.

### Knockdown of HSPA8 by Small Interfering RNA

A specific small interfering RNA (siRNA) targeting the rats HSPA8 gene was chemically synthesized by HanYi Biosciences Inc (Guangzhou, China) and was transfected into cells using Lipofectamine 3000 transfection reagent following the manufacturer’s instructions (Thermo Fisher, UT, USA).The HSPA8 siRNA sequences are as follows: sense strand 5′- GCACAGGAAAGGAGAACAATTUUGUUCUCCUUUCCUGUGCTT −3′.

### Ethics approval

The study was conducted according to the guidelines of the Declaration of Helsinki, and approved by the Ethical Committee of Guangzhou Red Cross Hospital of Jinan University (2023-076-01). Informed consent was obtained from all subjects involved in the study.

### Animal experiment

Six- to eight-week-old male *Sprague-Dawley (SD) rats* were purchased from the Guangdong Provincial Medical Laboratory Animal Center (Foshan, China). The rats were randomly assigned to four groups (n = 6 per group): sham-operated group, Hulth group, Hulth + low-dose CS group (100 mg/kg/day), and Hulth + high-dose CS group (300 mg/kg/day). Prior to surgery, the rats were fasted for 8 hours. After body weight measurement, the rats were anesthetized via intraperitoneal injection of an anesthetic mixture of ketamine and xylazine (1:2 ratio) at a dosage of 300 μl/kg, followed by skin preparation.The sham group received skin and joint capsule incision followed by immediate suturing. Osteoarthritis was induced in Hulth and CS-treated groups using modified Hulth’s method [33]: A medial parapatellar incision was made, fascia dissected layer-by-layer, medial collateral ligament transected, anterior cruciate ligament identified and transected, and medial meniscus excised. Sham controls underwent joint cavity exposure without additional interventions prior to closure. The doses of CS (100 and 300 mg/kg/d) were selected: the lower dose approximates the clinically relevant equivalent [34], while the higher dose was based on its established efficacy in a rodent arthritis model [35]; Our selected doses are also similar to those employed in a recent study [36].Starting on postoperative day 1, CS groups received daily oral gavage of chondroitin sulfate dissolved in saline (100 mg/kg or 300 mg/kg), while sham and Hulth groups received equivalent saline placebo for 8 weeks before euthanasia. Rats were anesthetized with an appropriate dose of a ketamine-xylazine mixture (1:2) to achieve a deep surgical plane of anesthesia, as confirmed by the absence of response to a painful stimulus. All animals were subsequently euthanized by cervical dislocation while under deep anesthesia to ensure a humane endpoint.

### Histological and immunohistochemical staining

Rat knee joints were harvested for macroscopic imaging under a stereo microscope (Nikon SMZ18) (S1 Fig). Joint tissues were fixed in 4% paraformaldehyde (PFA, P0099, Beyotime) for 24 hours, followed by decalcification in 10% EDTA (G1105, Servicebio) over 4 weeks. Decalcified tissues were paraffin-embedded, sectioned, and stained with Safranin O/Fast Green (SOFG, G1053, Servicebio). Cartilage degeneration was evaluated using the Osteoarthritis Research Society International (OARSI) scoring system. For immunohistochemistry, dewaxed and rehydrated sections underwent blocking before overnight incubation with primary antibodies. Sections were then incubated for 1 hour with species-matched HRP-conjugated secondary antibodies, developed with DAB substrate, and counterstained with hematoxylin. Immunopositive signals were visualized under a light microscope (Nikon Eclipse Ci).

Primary antibody: COL2A1 (1:800, 28459–1-AP, Proteintech, MMP13 (1:50, 18165–1-AP, Proteintech, ACSL4 (1:50, 22401–1-AP, Proteintech, HSPA8 (1:50, 10654–1-AP, Proteintech, SLC7A11 (1:100, AF7992, Beyotime).

### Statistical analysis

Data are presented as the mean ± standard deviation (SD) of at least three independent biological replicates (n ≥ 3). Statistical analyses were performed using GraphPad Prism 8.0.2 (GraphPad Software). Statistically significant differences were evaluated by ordinary one-way analysis of variance (ANOVA) followed by Tukey’s post-hoc test for multiple comparisons. Fluorescence images were processed and analyzed using ImageJ. A P value less than 0.05 was considered significant, and is denoted as “#” for P < 0.05, “##” for P < 0.01, and “ns” for non-significant differences.

## Results

### 1.Fer-1 inhibits RSL3-induced chondrocyte ferroptosis

**Fig 1:**
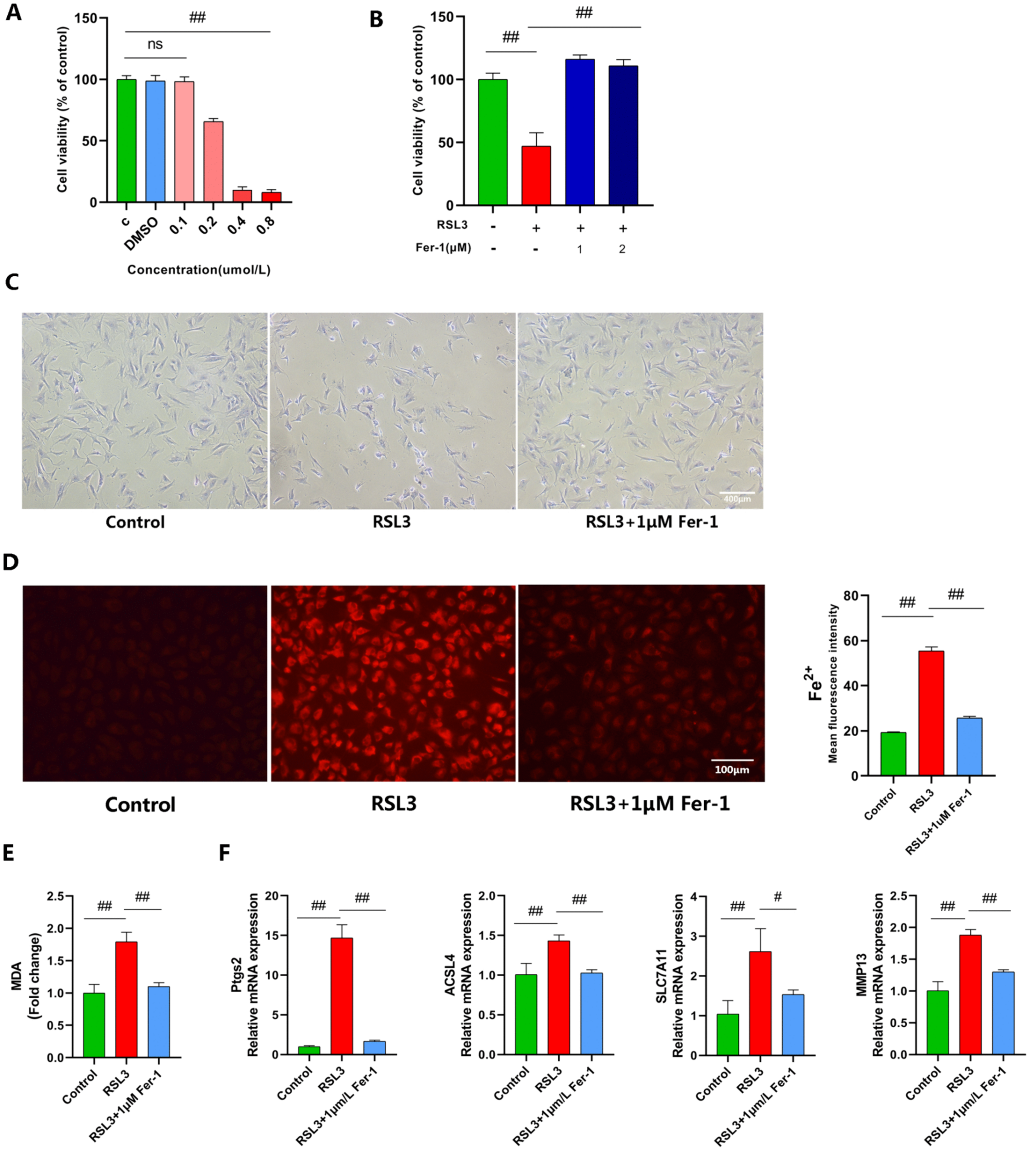
Fer-1 inhibits RSL3-induced chondrocyte ferroptosis. **(A, B)** Chondrocytes were treated with different concentrations of RSL3 (0.1, 0.2, 0.4, 0.8 μM) for 4 h (n = 3); or co-treated with different concentrations of Fer-1 (1, 2 μM) and 0.2 μM RSL3 for 4 **h.** Cell viability was determined by the CCK-8 assay (n = 3).**(C)** Morphology of chondrocytes was observed by Toluidine Blue staining (Scale bar, 400 μm).**(D)** Intracellular Fe² ⁺ levels were measured using Ferro Orange staining (n = 3; Scale bar, 100 μm).**(E)** Intracellular MDA content was measured using a commercial assay kit (n = 3).**(F)** The mRNA expression levels of ACSL4, Ptgs2, SLC7A11, and MMP13 were detected by RT-qPCR (n = 3) Data are presented as mean ± SD (n = 3). The “n” represents three independent biological replicates. #p < 0.05; ##p < 0.01.

**Fig 2:**
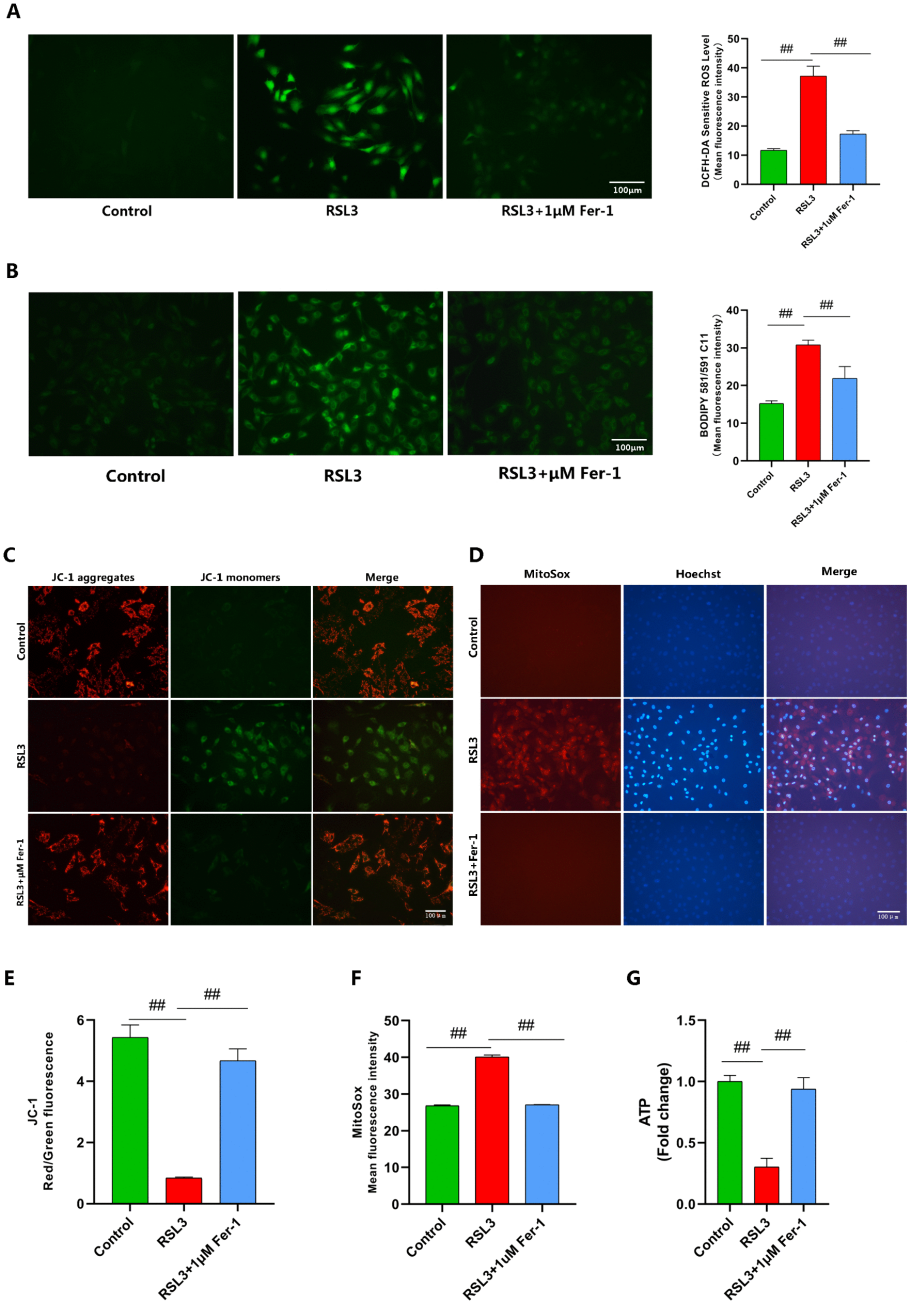
Fer-1 inhibits RSL3-induced chondrocyte ferroptosis. **(A, B)** Intracellular ROS levels were detected using the DCFH-DA fluorescent probe (n = 3; Scale bar: 100 µm). Intracellular LPO levels were detected using the BODIPY 581/591 C11 fluorescent probe (n = 3; Scale bar: 100 µm). **(C, E)** Mitochondrial membrane potential was assessed by JC-1 staining. The ratio of JC-1 red/green fluorescence intensity is shown (n = 3; Scale bar: 100 µm). **(D, F)** Mitochondrial ROS levels were measured by MitoSOX Red staining. MitoSOX mean fluorescence intensity is shown (n = 3; Scale bar: 100 µm). **(G)** Intracellular ATP content was measured (n = 3). Data are presented as mean ± SD (n = 3). The “n” represents three independent biological replicates.##p < 0.01.

We established a ferroptosis model using RSL3. Treatment with RSL3 (0.2–0.8 μM) for 4 hours significantly suppressed cellular viability (P < 0.05), with statistically significant differences observed (Fig 1A). Given that 0.2 μM RSL3 treatment for 4 hours induced approximately 60% inhibition of cellular viability, this concentration and duration were selected for subsequent experiments. Fer-1 a potent and selective ferroptosis inhibitor, was employed to validate the RSL3-induced ferroptosis model. Using CCK-8 assays and toluidine blue staining, we confirmed RSL3-mediated chondrocyte toxicity and demonstrated Fer-1’s mitigation of this cytotoxic effect (Fig 1B, C). Subsequent measurement of intracellular Fe² ⁺ levels using FerroOrange staining revealed significantly enhanced fluorescence signals in RSL3-treated chondrocytes compared to controls, indicating iron overload. Fer-1 treatment significantly attenuated these alterations (Fig 1D).Assessment of intracellular MDA levels revealed significantly elevated MDA production in RSL3-treated chondrocytes compared to controls. Conversely, Fer-1 treatment effectively suppressed MDA accumulation (Fig 1E). RT-qPCR analysis revealed significant upregulation of ACSL4, Ptgs2, and MMP13 in RSL3-treated chondrocytes versus controls, whereas Fer-1 attenuated these elevations (Fig 1F). SLC7A11 facilitates cystine transport into cells for GSH synthesis. Insufficient SLC7A11 expression reduces GSH production and promotes lipid peroxide accumulation, ultimately triggering ferroptosis [37]. Intriguingly, RSL3 treatment upregulated SLC7A11 expression compared to controls—a compensatory cellular response during early ferroptosis consistent with prior reports [22] (Fig 1F). Using DCFH-DA and BODIPY 581/591 C11 fluorescent probes to detect intracellular ROS and lipid LPO levels, fluorescence imaging revealed increased ROS and LPO in RSL3-treated chondrocytes, as indicated by enhanced green fluorescence intensity. In contrast, Fer-1 treatment significantly reduced ROS and LPO accumulation (Fig 2A, B).Mitochondrial damage occurs during ferroptosis, where defective mitochondria generate excessive ROS and deplete ATP reserves [38]. We therefore assessed ΔΨm, mtROS, and intracellular ATP levels using corresponding assay kits. As anticipated, Fer-1 restored ΔΨm alterations and attenuated ATP depletion while suppressing mtROS generation (Fig 2C-G).

In summary, Fer-1 validated the ferroptosis model established by RSL3, while concurrently confirming that RSL3 induces both OA-like changes and ferroptosis in chondrocytes. Critically, Fer-1 inhibited RSL3-triggered chondrocyte ferroptosis and associated OA-like pathological alterations.

### 2.CS inhibits RSL3-induced chondrocyte ferroptosis

**Fig 3:**
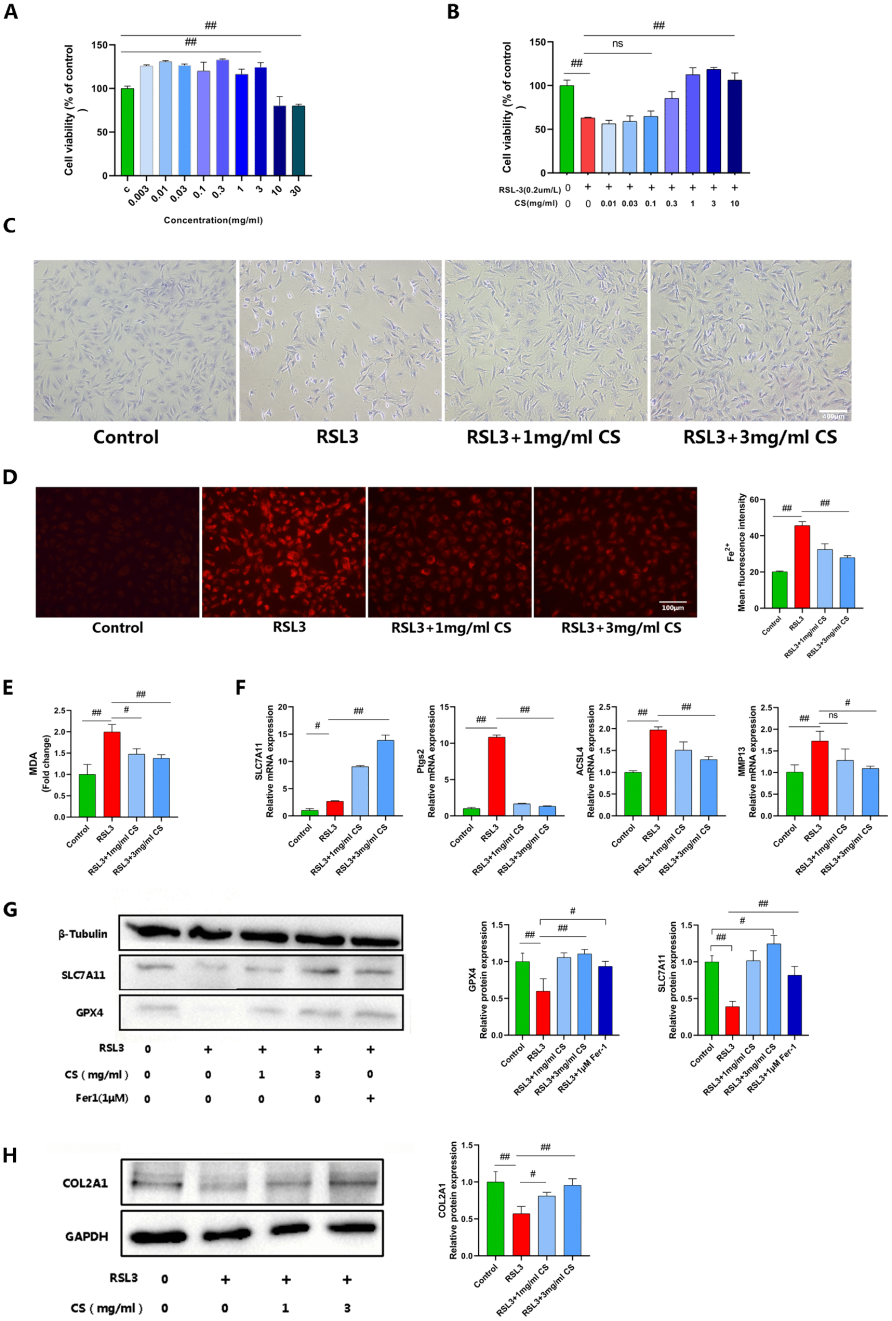
CS Inhibits RSL3-Induced Chondrocyte Ferroptosis. **(A, B)** Effects of different concentrations of CS on rat chondrocyte viability after 24 h treatment; Effects of co-treatment with different concentrations of CS and 0.2 μM RSL3 for 4 h on rat chondrocyte viability (n = 3).**(C)** Morphology of chondrocytes was observed by Toluidine Blue staining (Scale bar, 400 μm).**(D)** Intracellular Fe² ⁺ levels were measured using Ferro Orange staining (n = 3; Scale bar, 100 μm).**(E)** Intracellular MDA content was measured using a commercial assay kit (n = 3).**(F)** The mRNA expression levels of SLC7A11, Ptgs2, ACSL4, and MMP13 were detected by RT-qPCR (n = 3).**(G, H)** Protein expression levels of COL2A1, SLC7A11, and GPX4 were detected by Western blot (n = 3). Data are presented as mean ± SD (n = 3). The “n” represents three independent biological replicates. #p < 0.05; ##p < 0.01.

Chondrocyte viability was assessed via CCK-8 assay. Treatment with <3 mg/ml CS for 24 hours showed no significant toxicity, whereas ≥10 mg/ml CS significantly suppressed cellular viability (P < 0.05) (Fig 3A). Co-treatment with varying CS concentrations (0.1–3 mg/ml) and 0.2 μM RSL3 for 4 hours demonstrated that CS dose-dependently attenuated RSL3-induced reduction in chondrocyte viability, with significant protection observed at 1 mg/ml and 3 mg/ml (Fig 3B). Toluidine blue staining revealed brightened, shrunken morphology and reduced cell density in RSL3-treated chondrocytes versus controls. In contrast, CS-treated groups (1 mg/ml and 3 mg/ml) maintained normal cellular morphology (Fig 3C). Accordingly, 1 mg/ml and 3 mg/ml CS were selected as therapeutic doses for subsequent experiments.

Ferroptosis was evaluated using chondrocyte biomarkers: expression levels of PTGS2, ACSL4, SLC7A11, and GPX4, along with MDA and Fe² ⁺ concentrations. OA progression was monitored via COL2A1 and MMP13 expression. FerroOrange staining demonstrated significantly enhanced red fluorescence in RSL3-treated chondrocytes versus controls, while CS treatment attenuated this fluorescence intensification(Fig 3D). Similarly, CS downregulated elevated MDA levels (Fig 3E). RT-qPCR analysis revealed a consistent upregulation of ACSL4, MMP13, PTGS2, and SLC7A11 in RSL3-treated chondrocytes compared to controls.. CS significantly downregulated ACSL4, MMP13, and PTGS2 elevations. Intriguingly, CS dose-dependently upregulated SLC7A11 expression compared to controls (Fig 3F). Western blot analysis demonstrated reduced expression of COL2A1, SLC7A11, and GPX4 in RSL3-treated chondrocytes versus controls. Both Fer-1 and CS restored COL2A1 and GPX4 expression to baseline levels. Notably, CS significantly upregulated SLC7A11 expression compared to control groups (Fig 3G, H). These results demonstrate that CS inhibits chondrocyte ferroptosis while simultaneously alleviating RSL3-induced osteoarthritis-like pathological alterations.

**Fig 4:**
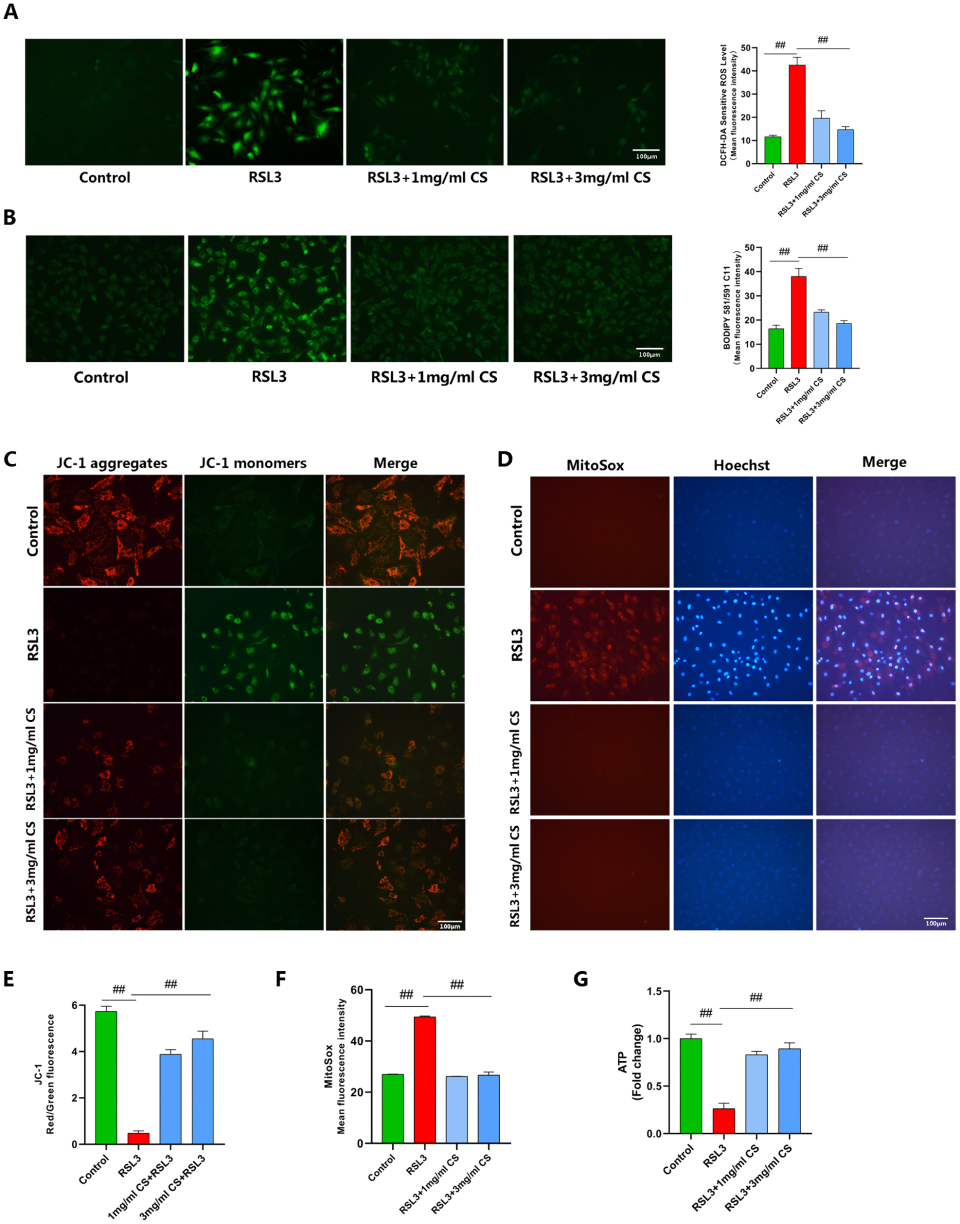
CS Inhibits RSL3-Induced Chondrocyte Ferroptosis. **(A, B)** Intracellular ROS levels were detected using the DCFH-DA fluorescent probe (n = 3; Scale bar: 100 µm). Intracellular LPO levels were detected using the BODIPY 581/591 C11 fluorescent probe (n = 3; Scale bar: 100 µm). **(C, E)** Mitochondrial membrane potential was assessed by JC-1 staining. The ratio of JC-1 red/green fluorescence intensity is shown (n = 3; Scale bar: 100 µm).**(D, F)** Mitochondrial ROS levels were measured by MitoSOX Red staining. MitoSOX mean fluorescence intensity is shown (n = 3; Scale bar: 100 µm).**(G)** Intracellular ATP content was measured (n = 3). Data are presented as mean ± SD (n = 3). The “n” represents three independent biological replicates. ##p < 0.01.

During ferroptosis, ROS accumulation triggers lipid peroxidation. We therefore detected intracellular ROS and LPO using DCFH-DA and BODIPY 581/591 C11 fluorescent probes. Fluorescence imaging revealed significantly increased ROS and LPO in RSL3-treated chondrocytes, as evidenced by enhanced green fluorescence intensity, while CS treatment markedly attenuated these elevations (Fig 4A, B).

Mitochondria play roles in diverse cell death mechanisms, including propagation of ferroptosis signaling. Thus, we assessed mitochondrial membrane potential using JC-1 staining, measured mtROS via MitoSOX Red staining, and quantified intracellular ATP levels with an ATP assay kit.Results demonstrated that compared to controls, RSL3-treated chondrocytes exhibited enhanced green fluorescence with significantly reduced red fluorescence in JC-1 staining, resulting in decreased red-to-green fluorescence ratio, along with elevated mtROS levels (evidenced by intensified red MitoSOX fluorescence) and reduced intracellular ATP content. Conversely, CS treatment prevented the RSL3-induced decline in JC-1 red-to-green fluorescence ratio and attenuated MitoSOX red fluorescence enhancement (Fig 4C-F), moreover restoring intracellular ATP levels (Fig 4G).

### 3.CS inhibition of chondrocyte ferroptosis may be associated with upregulation of HSPA8

The 2D and 3D molecular structures of CS were retrieved from the NCBI database (S2A Fig). The 3D structural file was submitted to the integrated pharmacophore matching platform PharmMapper, identifying 299 potential CS target proteins. Targets were filtered by a fit score >0.6, yielding 123 high-probability candidates. These genes were imported into the STRING platform to generate a protein-protein interaction (PPI) network (S2B Fig).GO enrichment analysis of the top 10 biological processes (BP), cellular components (CC), and molecular functions (MF) for CS target proteins revealed the most significantly enriched terms: purine-containing compound metabolic process, vesicle lumen, and endopeptidase activity. Results were visualized via bar plots and bubble plots (S3A, B Fig). To further investigate the potential pathways associated with CS target genes, we performed KEGG enrichment analysis on these targets. The results revealed significant enrichment of the target genes in the cysteine and glutathione metabolism pathway (S3C, D Fig). This aligns with the previously described fact that the amino acid transport system Xc ⁻ , a cystine/glutamate antiporter, influences cystine uptake. Disruption of system Xc⁻ leads to depletion of cysteine (Cys) and glutathione (GSH), thereby inducing ferroptosis. Osteoarthritis transcriptomic data were obtained from the GEO database (https://www.ncbi.nlm.nih.gov/geo/). After searching gene expression datasets with the filters series type and expression profiling by array, and excluding non-human specimens, dataset GSE114007 was selected. Differential gene expression analysis using the limma package in R identified 1,044 differentially expressed genes (DEGs), comprising 539 downregulated and 505 upregulated genes. The top 50 DEGs were visualized via a heatmap generated with the pheatmap function, while a volcano plot was created using the ggplot2 package (S4A, B Fig). Ferroptosis-associated targets were identified from the GeneCards database (n = 1,316) and the specialized ferroptosis database FerrDb V3 (n = 1,256). Using R software, osteoarthritis differentially expressed genes (OA DEGs), CS-targeted genes, and ferroptosis-associated targets from both sources were integrated. The VennDiagram package generated an intersection plot, specifically identifying HSPA8 as the overlapping gene (S4C Fig).

**Fig 5:**
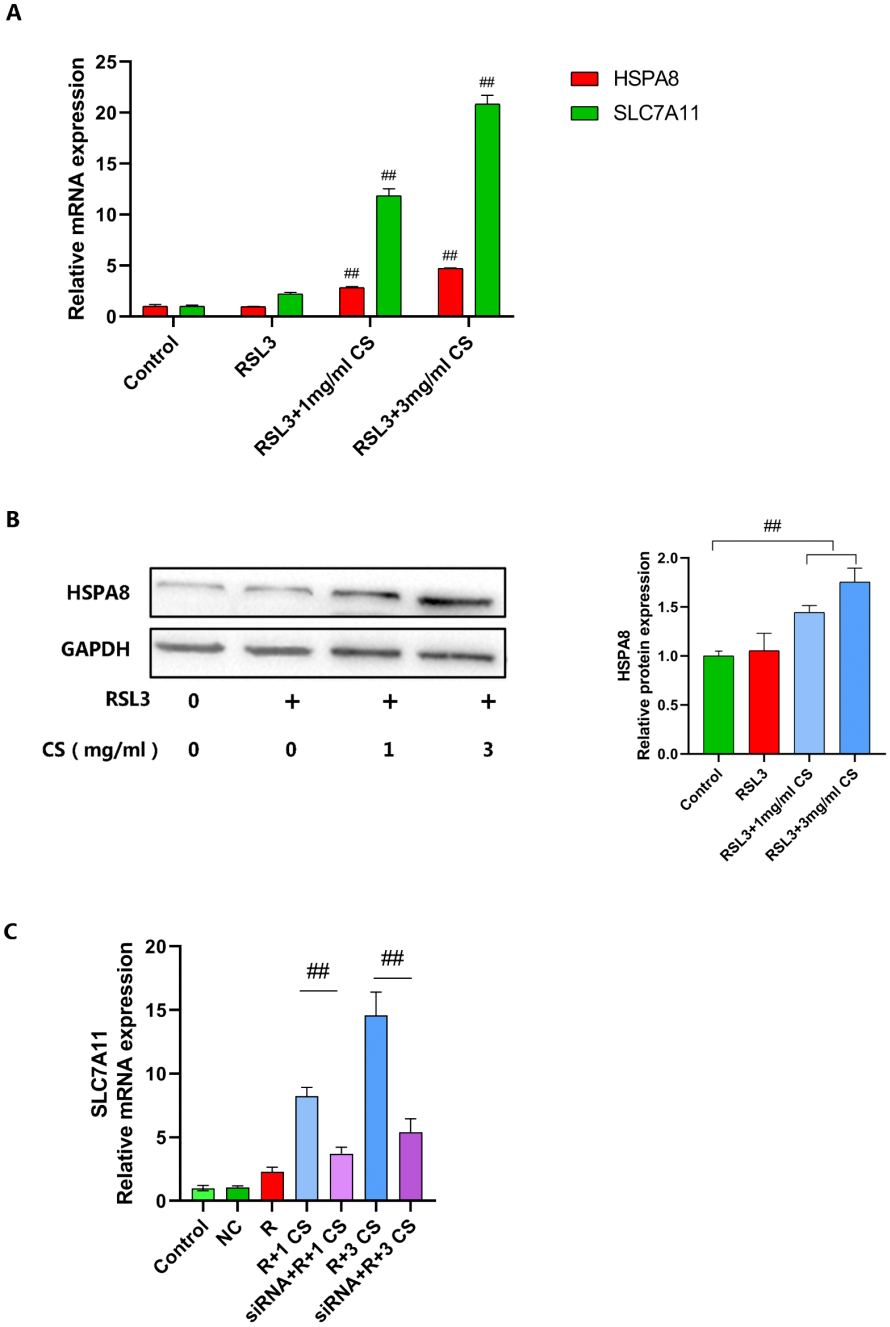
CS inhibition of chondrocyte ferroptosis may be associated with upregulation of HSPA8. **(A)** The mRNA expression levels of HSPA8 and SLC7A11 were detected by RT-qPCR (n = 3).**(B)** The protein expression level of HSPA8 was detected by Western blot (n = 3). **(C)** The mRNA expression levels of SLC7A11 were detected by RT-Qpcr (n = 3). NC: Negative Control, R:RSL3, 1 CS: 1 mg/ml CS, 3 CS: 3 mg/ml CS. Data are presented as mean ± SD (n = 3). The “n” represents three independent biological replicates.##p < 0.01.

HSPA8 localizes to the plasma membrane, cytoplasm, and nucleus, regulating cellular proteostasis by mediating protein assembly, refolding, transport, and degradation [39]. Notably, elevated HSPA8 expression upregulates SLC7A11 in the system Xc⁻ to inhibit ferroptosis [40]—a finding consistent with our experimental observation of heightened SLC7A11 expression. To validate whether CS upregulates SLC7A11 expression via HSPA8 induction, we assessed HSPA8 levels post-CS intervention using RT-qPCR (Fig 5A) and Western blot (Fig 5B). As hypothesized, CS-treated chondrocytes exhibited significantly elevated HSPA8 and SLC7A11 expression versus controls, demonstrating dose dependency. To further elucidate the relationship between HSPA8 and SLC7A11, HSPA8 was silenced using siRNA. A robust knockdown was achieved, as evidenced by RT-qPCR, showing about an 80% decrease in HSPA8 expression compared to the control cells(S5 Fig). Furthermore, following HSPA8 silencing, SLC7A11 expression was significantly downregulated across different concentrations of CS treatment compared to the non-silenced group (Fig 5C).These results suggest that CS inhibits RSL3-induced ferroptosis by enhancing HSPA8-mediated upregulation of SLC7A11 and GPX4 in chondrocytes.

### 4.CS alleviates articular cartilage inflammation and improves ferroptosis-related indicators

**Fig 6:**
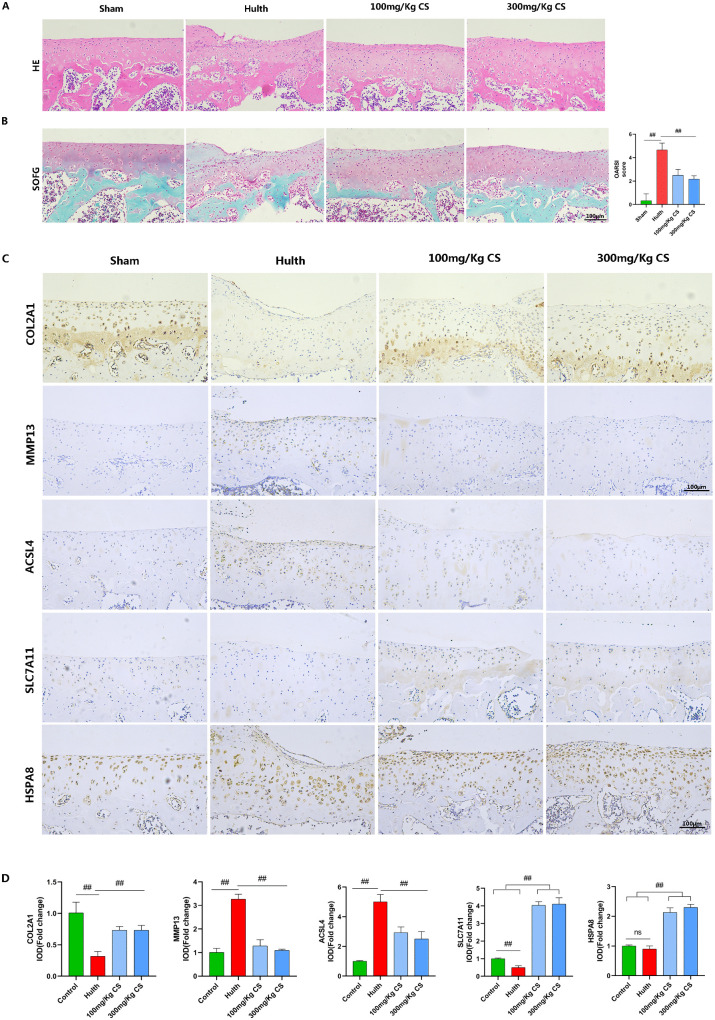
CS alleviates articular cartilage inflammation and improves ferroptosis-related indicators. **(A)** Hematoxylin and eosin (H&E) staining of rat knee joints.**(B)** Safranin O-Fast Green staining and OARSI scoring of rat knee joints (n = 3; Scale bar: 100 µm).**(C, D)** Expression and statistical analysis of COL2A1, MMP13, ACSL4, SLC7A11, and HSPA8 in rat knee articular cartilage (n = 3; Scale bar: 100 µm). Data are presented as mean ± SD (n = 3). The “n” represents three independent biological replicates. ##p < 0.01.

To investigate the protective effects of CS on OA progression, we induced OA in rats using modified Hulth’s surgery. OA severity was evaluated via OARSI scoring. Hematoxylin-eosin (HE) and safranin O-fast green (SOFG) staining revealed significant cartilage damage in the Hulth group. In contrast, both low-dose (100 mg/kg/day) and high-dose (300 mg/kg/day) CS-treated Hulth groups exhibited smooth, intact cartilage surfaces with significantly lower OARSI scores (P < 0.05) (Fig 6A, B). Furthermore, immunohistochemical analysis of OA markers revealed significantly reduced COL2A1 and markedly upregulated MMP13 expression in the Hulth model group versus the Sham group. CS treatment restored COL2A1 levels and prevented MMP13 upregulation (Fig 6C, D). Immunohistochemical analysis of ferroptosis markers revealed significantly upregulated ACSL4 and downregulated SLC7A11 expression in the Hulth model group versus Sham controls. Conversely, CS treatment reversed these alterations. Consistent with in vitro findings, CS-treated groups exhibited dose-dependent upregulation of SLC7A11 and HSPA8 (Fig 6C, D). These results indicate that CS likely inhibits chondrocyte ferroptosis and mitigates osteoarthritis progression by upregulating SLC7A11 expression through HSPA8 induction.

## Discussion

Ferroptosis, a regulated form of cell death discovered a decade ago, was initially described by Dixon [14]. In recent years, the role of ferroptosis in OA pathogenesis has attracted significant attention. Studies reveal that certain pathological features of OA resemble ferroptosis characteristics, including lipid peroxidation [41] and mitochondrial dysfunction [42]. A recent investigation by Yao et al. demonstrated that chondrocyte ferroptosis actively promotes OA initiation and progression [24]. Existing evidence indicates that CS exhibits anti-inflammatory, antioxidant, and anti-apoptotic properties in chondrocytes [27–32]. Nevertheless, whether CS can inhibit chondrocyte ferroptosis or attenuate OA progression remains unclear.

Our findings demonstrate that RSL3-induced ferroptosis promotes OA-like pathological changes in chondrocytes, specifically inflammation, oxidative stress, and ECM degradation..Conversely, the ferroptosis inhibitor Fer-1 inhibited both ferroptosis in chondrocytes and the RSL3-induced osteoarthritis (OA)-like changes in these cells. This indicates that suppressing ferroptosis in chondrocytes can alleviate chondrocyte inflammation and matrix degradation. Similarly, our study demonstrated that CS effectively reversed the elevated levels of ACSL4, MMP13, and Ptgs2, and reduced intracellular ROS and LPO levels in chondrocytes.Furthermore, CS significantly upregulated SLC7A11 expression, and notably, this upregulation exhibited a dose-dependent relationship with CS treatment.Importantly,CS reversed mitochondrial damage induced by ferroptosis and attenuated the ferroptosis-associated increases in MDA and Fe² ⁺ . Collectively, our findings demonstrate that CS protects chondrocytes by inhibiting ferroptosis, thereby delaying the progression of OA.

Heat shock proteins (HSPs) comprise a large family of structurally conserved molecular chaperones activated under thermal stress. More broadly, they respond to diverse environmental, physical, and chemical stressors—including cold exposure, UV radiation, and tissue damage—by rapidly expressing to mitigate stress-induced cellular injury and promote recovery. The heat shock protein 70 (HSP70) family represents ubiquitously expressed chaperones with at least thirteen members, including HSPA8 [43]. Existing studies have demonstrated that HSPA8 plays pivotal roles in various biological processes through the regulation of distinct downstream targets. For example, in cancer contexts, it not only modulates VAV1 to influence the ERK pathway [44] but also regulates BCR-ABL to impact drug resistance in leukemia [45]; in stem cell biology, HSPA8 maintains pluripotency by binding to and stabilizing OCT4 [46].As previously mentioned, the expression of SLC7A11 is more directly associated with system Xc⁻ activity, and inhibition of system Xc⁻ function induces ferroptosis.In this study, we found that treatment with CS significantly increased the expression of HSPA8 and SLC7A11 in chondrocytes, and this effect was dose-dependent. Importantly, HSPA8 knockdown abolished the CS-induced upregulation of SLC7A11, demonstrating that CS requires HSPA8 to modulate SLC7A11 expression.These results suggest that CS may inhibit RSL3-induced ferroptosis by modulating HSPA8-mediated upregulation of SLC7A11 and GPX4 expression in chondrocytes.

To investigate the potential protective role of CS in OA development, we induced OA in rats using the modified Hulth method. Histological analysis with Safranin O-Fast Green and H&E staining demonstrated that CS treatment mitigated cartilage defects and degeneration. Specifically, CS intervention reduced OA-associated loss and destruction of the cartilage matrix. Consequently, CS administration significantly reduced the OARSI scores in the OA model rats.During the progression of OA, COL2A1 and matrix MMP13 serve as crucial biomarkers. MMP13, a collagen-degrading metalloproteinase, demonstrates altered expression levels under inflammatory conditions, serving as an indicator of cartilage tissue integrity [47].Furthermore, numerous studies have utilized changes in the expression of GPX4, SLC7A11, and ACSL4 as biomarkers of ferroptosis [19,22]. Therefore, in this study, immunohistochemical analysis employed COL2A1 and MMP13 as OA markers, and SLC7A11 and ACSL4 as ferroptosis markers.This study found that CS treatment alleviated inflammation and suppressed ferroptosis in the articular cartilage of rats. Consistent with in vitro cellular experiments, SLC7A11 and HSPA8 expression levels were significantly upregulated in the CS-treated groups compared to other groups, and this upregulation was dose-dependent on CS. Therefore, we propose that the mechanism by which CS suppresses ferroptosis may involve the upregulation of HSPA8 and SLC7A11, a key ferroptosis-related gene. However, this study has several limitations. First, we did not further validate the HSPA8-associated pathway through gene knockout experiments in rats. Second, given CS’s complex pharmacokinetics as a polysaccharide and its ability to extensively modulate pathways—such as directly inhibiting NF-κB nuclear translocation and inflammation in chondrocytes via binding to TLR4, CD44, and ICAM-1 [48] —the underlying mechanisms of CS beyond these known actions warrant further exploration.In summary, our study demonstrates through in vitro and in vivo experiments that CS exerts protective effects against OA by suppressing ferroptosis in chondrocytes. This mechanism may involve the upregulation of HSPA8 and the key ferroptosis-related gene SLC7A11. These findings provide a novel theoretical foundation for understanding the mechanism of CS action and developing therapeutic strategies for OA.

## Supporting information

S1 Fig Representative photographs of rat knee joints. (TIF)

S2 Fig (A) CS Molecular Structure Visualization Chart. (B) PPI Network Map.
(TIF)

S3 Fig (A, B) GO Enrichment Analysis Chart. (C, D) KEGG Enrichment Analysis Chart.
(TIF)

S4 Fig (A, B) OA Differential Genes. (C) Venn Diagram.
(TIF)

S5 Fig The knockdown efficiency of siRNA targeting HSPA8. (TIF)

S1 Table Primers information (5′–3′).
(PDF)

S1 Data Raw data. (XLSX)
