# Supplementary material for: Chondroitin sulfate alleviates osteoarthritis by upregulating HSPA8 to inhibit chondrocyte ferroptosis
Source: PLoS One. 2026 Feb 19;21(2):e0342242. doi: 10.1371/journal.pone.0342242 (PMC12919810; doi:10.1371/journal.pone.0342242)
Supplement: S1 Table — Primers information (5′–3′). (PDF) [file pone.0342242.s006.pdf]

S1 Table . Primers information (5′–3′)

Gene	5′ - 3′
GAPDH	F:GGGCTGGCATTGCTCTCAA
	R:GTATCCTTGCTGGGCTGGG
ACSL4	F:CCCCAGACACACCGATTCAT
	R:GAGCGCCAACTCTTCCAGTA
Ptgs2	F:GATGACGAGCGACTGTTCCA
	R:TGGTAACCGCTCAGGTGTTG
MMP13	F:AGAAGTGTGACCCAGCCCTA
	R:GGTGCAGACGCCAGAAGA
HSPA8	F:GCAATGAACCCCACCAACAC
	R:TGCATCGTTCACCACCATGA
SLC7A11	F:ACAAGAAACCCAAGTGGTTCA
	R:TTTTGTATCTCAGTCCTGGGCA
